# Genome Sequence of the Mycorrhizal Helper Bacterium *Pseudomonas fluorescens* BBc6R8

**DOI:** 10.1128/genomeA.01152-13

**Published:** 2014-01-09

**Authors:** A. Deveau, H. Gross, E. Morin, T. Karpinets, S. Utturkar, S. Mehnaz, F. Martin, P. Frey-Klett, J. Labbé

**Affiliations:** INRA, Interactions Arbres – Microorganismes, UMR1136, Champenoux, Francea; Université de Lorraine, Interactions Arbres – Microorganismes, UMR1136, Vandœuvre-lès-Nancy, Franceb; Pharmaceutical Institute, Department of Pharmaceutical Biology, University of Tübingen, Tübingen, Germanyc; Biosciences Division, Oak Ridge National Laboratory, Oak Ridge, Tennessee, USAd; Graduate School of Genome Science and Technology, University of Tennessee, Knoxville, Tennessee, USAe; Department of Biological Sciences, Forman Christian College University, Lahore, Pakistanf

## Abstract

We report the draft genome sequence of the mycorrhizal helper bacterium *Pseudomonas fluorescens* strain BBc6R8. This is the first genome of a mycorrhizal helper bacterium. The draft genome contains 6,952,353 bp and is predicted to encode 6,317 open reading frames. Comparative genomic analyses will help to identify helper traits.

## GENOME ANNOUNCEMENT

*Pseudomonas fluorescens* BBc6R8 is a Gram-negative rod-shaped bacterium isolated from a sporocarp of the ectomycorrhizal fungus *Laccaria bicolor* (1), and it is characterized by its abilities to improve mycorrhizal formation and to stimulate the hyphal growth and survival of the ectomycorrhizal fungus *L. bicolor* S238N (2, 3), a model organism for mycorrhizal formation. Despite the ecological importance of helper bacteria in the functioning of the forest ecosystem and their potential applications for sustainable tree production in nurseries, little is known about the mechanisms underlying the helper phenotype. To gain insight into the mechanisms of the helper effect as well as the ecological traits of the strain BBc6R8, we acquired the complete genome sequence of the strain.

The draft genome sequence data of *P. fluorescens* BBc6R8 were generated by using a combination of 454 and Illumina HiSeq 2000 technologies from 3-kb mate-pair and 500-bp paired-end libraries, respectively. The reads were assembled into 153 contigs and 5 scaffolds using Newbler software (4). Gene calling and annotation were first performed automatically using the IMG/ER (5) pipelines, followed by metabolic reconstruction via PathwayTool version 16.5 pipeline (6) and manual curation. The draft genome sequence consists of approximately 7 Mb, with an overall G+C content of 61%, and contains 6,317 predicted genes, 4 RNA operons, and 57 tRNA loci. Although the strain belongs to subgroup V of the *P. fluorescens* species, the genome shows a relatively low level of synteny and orthology with those of other *P. fluorescens* strains from the same subgroup, e.g., strains SBW25 and WH6, which have only 73 and 71 percent orthologous sequences, respectively. More detailed genomic analyses and comparative studies with other *P. fluorescens* genomes will give insights into the mechanisms of the mycorrhizal helper effect.

### Nucleotide sequence accession numbers.

The nucleotide sequence has been deposited at DDBJ/EMBL/GenBank under the accession no. AKXH00000000, and the version described in this paper is version AKXH02000000.
